# Neutrophil-lymphocyte, platelet-lymphocyte and lymphocyte-monocyte ratios may not be useful markers to assess disease activity in rheumatoid arthritis: A STROBE-compliant article

**DOI:** 10.1097/MD.0000000000027631

**Published:** 2021-11-12

**Authors:** Wang Lijuan, Zhou Yuting, Liang Chaoyang, Yang Ju

**Affiliations:** a Chengdu Medical College, Chengdu, China; b North Sichuan Medical College, Nanchong, China; c Department of Rheumatology, The First People's Hospital of Yibin, Yibin, China.

**Keywords:** lymphocyte-monocyte ratio., neutrophil-lymphocyte ratio, platelet-lymphocyte ratio, rheumatoid arthritis

## Abstract

The associations among the neutrophil-lymphocyte ratio (NLR), platelet-lymphocyte ratio (PLR) and lymphocyte-monocyte ratio (LMR) and disease activity in rheumatoid arthritis remains unclear.

To evaluate these indicators as potential markers of disease activity in patients with rheumatoid arthritis (RA).

This cross-sectional study included 547 adult patients with RA. The patients were divided into two groups according to the disease activity score (DAS) system: remission and disease activity. Differences in the NLR, PLR and LMR of the two groups were assessed. Correlations were analyzed using Spearman analysis, and receiver operating characteristic (ROC) curves were used to identify the sensitivity, specificity, and optimal cutoff values to differentiate active RA patients from inactive RA patients.

There was a statistically significant difference in the NLR (4.2 ± 3.2 vs 3.4 ± 2.4, *P* = .034) and PLR (222.3 ± 136.4 vs 176.9 ± 89.8, *P* = .006) between the two groups, but not for the LMR (3.0 ± 1.8 vs 3.4 ± 2.4, *P* = .115). In addition, the DAS28 and traditional inflammatory markers, including ESR and CRP, were weakly positively correlated with the NLR and PLR. Based on the ROC curves, the NLR (sensitivity 31.8%, specificity 77.8%) and PLR (sensitivity 57.3%, specificity 63.9%) were less valuable than the ESR (sensitivity 67.2%, specificity 91.7%) and CRP (sensitivity 76.2%, specificity 91.7%) for differentiating inactive RA patients from active RA patients due to low sensitivity and specificity and combining NLR or PLR also cannot significantly improved the diagnostic value of ESR and CRP.

NLR, PLR and LMR may not be an useful independent diagnostic or complementary marker for disease activity in RA patients.

## 1 Introduction

Rheumatoid arthritis (RA) is a systemic autoimmune rheumatic disease that affects synovial joints, leading to bone damage, disability and excess cardiovascular events and cardiovascular mortality.^[1–3]^ Inflammation is the key component of RA pathology. Thus, accurate measurement of inflammatory activity is essential for customizing the treatment strategy. Although the erythrocyte sedimentation rate (ESR), C-reactive protein (CRP), and disease activity score (DAS) are currently used to estimate RA, several studies have reported the limitations of these markers, such as flooring effects at lower disease activity.^[4–6]^ Ultrasound and magnetic resonance imaging have been reported to show synovial inflammation, even when the ESR, CRP and DAS have reached the lowest or near normal levels.^[7,8]^ However, these techniques are expensive and time consuming.

The neutrophil-lymphocyte ratio (NLR), in addition to the platelet-lymphocyte ratio (PLR) and lymphocyte-monocyte ratio (LMR), is a simple biomarker of systemic inflammation and has been reported to be a highly sensitive measure of inflammation in the field of oncology (i.e., gastric cancer,^[9]^ breast cancer,^[10]^ prostate cancer^[11]^ and non-small-cell lung cancer^[12]^, cardiology,^[13]^ diabetes^[14]^ and infectious diseases).^[15]^ Recently, several studies have reported significant associations among the NLR, PLR, LMR and the presence of RA, as well as disease activity.^[16–22]^

Accordingly, the aim of the present study was to determine the relationships among the NLR, PLR, and LMR levels and disease activity in RA. In addition, this study aimed to correlate the NLR and PLR with the inflammatory markers ESR, CRP and DAS28 and to evaluate the ability of the NLR and PLR to discriminate between patients with active RA and those in remission.

## 2 Materials and methods

### 2.1 Patients and study design

This was a single-center, cross-sectional study performed in the First People's Hospital of Yibin. The electronic records of patients with RA who were treated at the Department of Rheumatology between 2018 and 2019 were retrospectively reviewed. RA was diagnosed in accordance with the guidelines developed by the American College of Rheumatology (ACR)/European League Against Rheumatism (EULAR) in 2010. Individuals who had any conditions that may affect the NLR, PLR and LMR values were excluded, such as use of steroid at a dose of more than 7.5 mg/day of prednisone or equivalent, active infection, malignancy, or other autoimmune diseases. Age, sex, duration of illness, visual analog scale (VAS) score for pain, swollen joint count (SJC)-28, tender joint count (TJC)-28, and laboratory results for white blood cell count, neutrophil count, lymphocyte count, platelet count, monocyte count, CRP and ESR were recorded. The NLR and PLR for each participant were calculated manually by dividing the neutrophil count and platelet count, respectively, by the lymphocyte count after obtaining the laboratory results. The LMR was defined as the lymphocyte count divided by the monocyte count. The disease activities of patients with RA were determined by the Disease Activity Score of 28 joints (DAS28) system, which was measured by calculating the number of tender joints and swollen joints, the ESR or CRP, and the patient global health assessment using a visual analog scale. Accordingly, the disease can be categorized as the severe activity group (DAS28 ≥ 5.1), moderate activity group (3.2 ≤ DAS28 < 5.1), low activity group (2.6 ≤ DAS28 < 3.2) and remission (< 2.6). We divided the patients into two groups according to the DAS28 system. Group A included patients with a score lower than 2.6 by the DAS28 system (patients in remission), and Group B included patients with a score of 2.6 and higher (patients with active disease). The study protocol was approved by the Institutional Review Board of Chengdu Medical College in accordance with the Declaration of Helsinki, and patient approval or informed consent was required for our review of the patients’ medical records.

### 2.2 Statistical analyses

Statistical analyses were conducted using SPSS version 22.0 (SPSS Inc., Chicago, IL, USA), and all the graphics were plotted with GraphPad Prism 8.0 (GraphPad Prism Software Inc., San Diego, CA, USA). The normality of distribution was assessed using the Shapiro-Wilk test. Continuous variables are expressed as the mean ± SD, while categorical variables are presented in absolute numbers and percentages (n [%]), and the differences were compared and analyzed using Student's sample *t* test, chi-squared test, or Fisher's exact test. Spearman correlation was conducted to evaluate the linear relationship. The receiver operating characteristic (ROC) curve was used to assess the discriminative ability for RA disease activity. The area under the curve (AUC), optimal cutoff values, specificity and sensitivity were also determined. A two-sided *P* value of < .05 was regarded as statistically significant.

## 3 Results

### 3.1 Patient characteristics and laboratory data

The detailed characteristics of RA patients are summarized in Table 1. A total of 547 patients with RA were enrolled in our study, including 72 patients in remission and 475 patients with active disease. No significant differences were observed between the two groups in sex composition, body mass index, disease duration or lymphocytes (all *P* > .05). Age (*P* = .038) and hemoglobin (*P* < .001) were significantly higher in the remission group than in the disease activity group. However, the other indicators, such as doses of methotrexate, white blood cells, neutrophils, monocytes, platelets, rheumatoid factor, ESR and CRP, were significantly lower in the remission group than in the disease activity group (all *P* < .05).

**Table 1:** Baseline demographics and and hematologic parameters of RA patients according to DAS-28.

		Disease activity according to DAS-28		
Characteristics	All RA patients (n = 547)	RA in remission (n = 72)	RA in activity (n = 475)	*P* Value
Age (years)	55.8 ± 11.9	58.5 ± 11.1	55.4 ± 12.0	***0.038*** ^∗^
Gender (Male/Female), n (%)	102 (18.6)/445 (81.4)	8 (11.1)/64 (88.9)	94 (19.8)/381 (80.2)	0.078
Body mass index (Kg/m^2^)	22.7 ± 3.3	22.8 ± 3.1	22.6 ± 3.3	0.800
Disease duration, years, median (range)	9 (0–43)	9 (0–42)	10 (0–43)	0.472
Leflunomide dose in mg/day, median (range)	10 (0–20)	10 (0–20)	10 (0–20)	0.107
Methotrexate dose in mg/week, median (range)	0 (0–70)	0 (0–10)	10 (0–70)	***<0.001*** ^∗^
Hydroxychloroquine dose in g/day, median (range)	0 (0–0.6)	0 (0–0.6)	0 (0–0.6)	0.211
Prednisone dose in mg/day, median (range)	0 (0–6)	0 (0–6)	0 (0–6)	0.188
Hemoglobin (g/L)	112.1 ± 20.0	121.1 ± 17.3	110.7 ± 20.0	***<0.001*** ^∗^
White blood cell (10^9^/L)	5.7 ± 2.8	5.4 ± 2.2	5.8 ± 1.6	***<0.001*** ^∗^
Neutrophils (10^9^/L)	4.7 ± 2.5	3.6 ± 1.7	4.9 ± 2.6	***<0.001*** ^∗^
Lymphocytes (in 10^9^/L)	1.4 ± 0.7	1.3 ± 0.7	1.4 ± 0.7	0.353
Monocytes (10^9^/L)	0.4 ± 0.5	0.2 ± 0.3	0.5 ± 0.5	***<0.001*** ^∗^
Platelet (10^9^/L)	248.5 ± 111.0	191.5 ± 75.9	257.2 ± 111.5	***<0.001*** ^∗^
rheumatoid factor (IU/mL)	244.9 ± 260.2	204.0 ± 260.0	251.2 ± 260.0	***<0.001*** ^∗^
Erythrocyte sedimentation rate (mm/h)	52.7 ± 36.2	19.9 ± 15.7	57.7 ± 35.9	***<0.001*** ^∗^
reactive protein (mg/L)	31.7 ± 41.5	3.1 ± 3.2	36.0 ± 42.9	***<0.001*** ^∗^
Visual analogue scale score	3.1 ± 2.0	0.3 ± 0.8	3.5 ± 1.8	***<0.001*** ^∗^
Swollen joint count-28, median (range)	14 (0–28)	0 (0–2)	18 (0–28)	***<0.001*** ^∗^
Tender joint count-28, median (range)	16 (0–28)	0 (0–2)	22 (0–28)	***<0.001*** ^∗^
DAS-28 ESR score	5.3 ± 2.0	2.2 ± 0.4	5.8 ± 1.6	***<0.001*** ^∗^
DAS class, n (%)				
Remission	72 (13.2)	72 (100.0)	0 (0.0)	***<0.001*** ^∗^
Low disease activity	56 (10.2)	0 (0.0)	56 (11.8)	
Moderate disease activity	97 (17.7)	0 (0.0)	97 (20.4)	
High disease activity	322 (58.9)	0 (0.0)	322 (67.8)	

DAS-28 = Disease Activity Score of 28 joints, RA = Rheumatoid arthritis.

∗ Statistically significant difference

### 3.2 Differences in the mean NLR, PLR and LMR between remission and active RA patients

**Figure 1:**
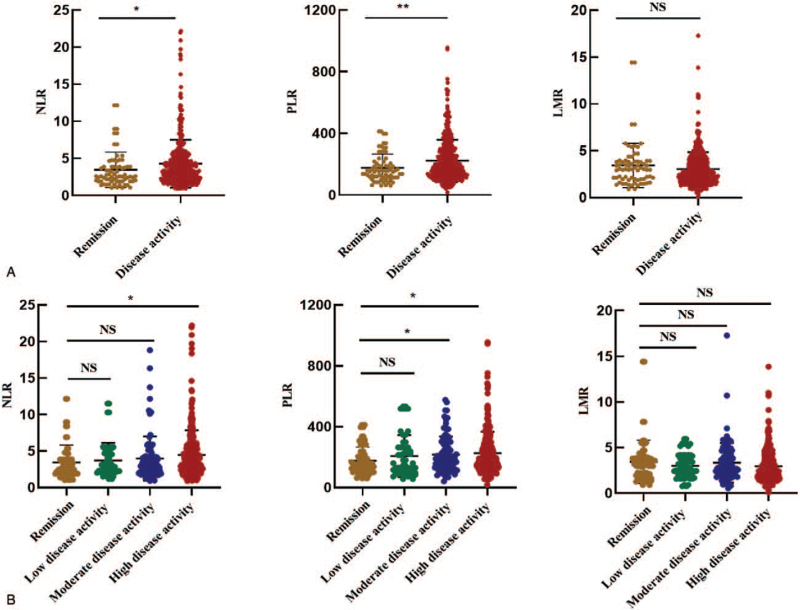
The mean values of the NLR, PLR and LMR in remission and active disease patients according to the DAS28.

As shown in Fig. 1A, the mean NLR (4.2 ± 3.2 vs 3.4 ± 2.4, *P* = .034) and PLR (222.3 ± 136.4 vs 176.9 ± 89.8, *P* = .006) in the disease activity group were significantly higher than those in the remission group. No significant differences were identified in the LMR between the two groups (3.0 ± 1.8 vs 3.4 ± 2.4, *P* = .115). Next, we further divided the patients with active RA into three subgroups, including low disease activity (n = 56), moderate disease activity (n = 97) and high disease activity (n = 322) (Table 1). Figure 1B demonstrates that the mean NLR in patients with high disease activity was significantly higher than that in patients in remission, but no significant differences were found in the low disease activity and moderate disease activity groups compared to patients in remission. Both the mean values of the PLR in patients with high disease activity and moderate disease activity were significantly higher than the values in patients in remission, but no significant differences were found in the low disease activity patients compared to patients in remission. Additionally, the mean values of the LMR in patients in remission were not significantly different from those in patients in the other three disease activity groups.

### 3.3 Correlation of NLR and PLR levels with laboratory data and ROC analysis

**Figure 2:**
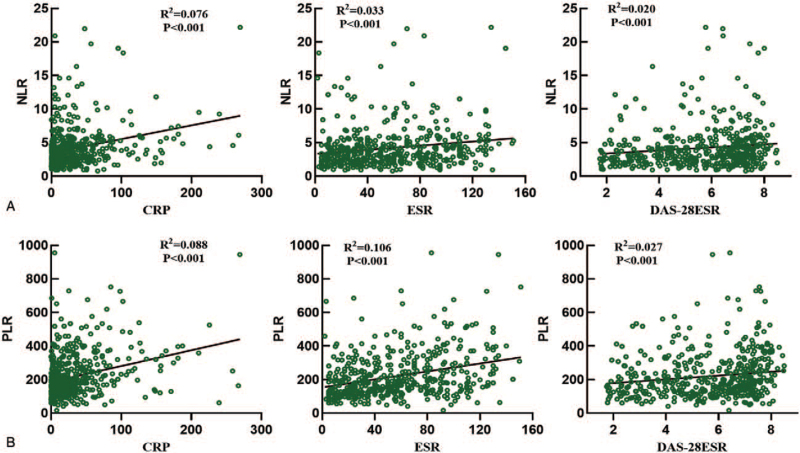
Correlation between the NLR and PLR and CRP, ESR, and DAS28 in patients with RA.

Analysis of the correlations of the NLR with CRP, ESR and DAS28-ESR, the three most extensively used parameters for RA disease activity assessment, showed that all these indices were weakly positively correlated with the NLR (R^2^ = 0.076, *P* < .001; R^2^ = 0.033, *P* < .001; and R^2^ = 0.020, *P* < .001, respectively) (Fig. 2A). Moreover, the data demonstrated that the PLR was weakly positively correlated with the abovementioned inflammatory markers (R^2^ = 0.088, *P* < .001; R^2^ = 0.106, *P* < .001; and R^2^ = 0.027, *P* < .001, respectively) (Fig. 2B).

**Figure 3:**
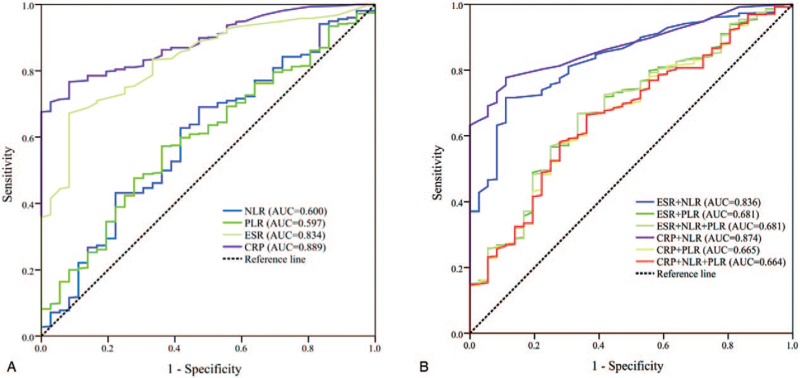
ROC curves of the NLR, PLR, ESR and CRP differentiating active RA patients from those in remission.

Furthermore, the NLR, PLR, CRP and ESR were valid fair tests to differentiate active RA from inactive RA by ROC analysis, where the AUCs for the NLR and PLR were 0.600 and 0.597, respectively, with *P* < .05. The optimum cutoff value for the NLR was ≥4.5 (sensitivity of 31.8%, specificity 77.8%) and 167.5 for the PLR (sensitivity 57.3%, specificity 63.9%). However, the AUC for the ESR was 0.834 with a cutoff value of ≥34.5, sensitivity of 67.2%, and specificity of 91.7%. The AUC for CRP was 0.889 with a cutoff value of ≥7.5, sensitivity of 76.2%, and specificity of 91.7%. These parameters are shown in Fig. 3A andTable 2. Additionally, combining NLR or PLR cannot significantly improved the diagnostic value of ESR and CRP (Fig. 3B and Table 2).

**Table 2:** ROC curve analysis and validity of NLR, PLR, ESR and CRP to differentiate between active and inactive RA patients.

Variables	AUC	95% CI	Cut-off value	Sensitivity	Specificity	You den index	*P* value
NLR	0.600	0.525–0.670	4.5	31.8%	77.8%	0.096	**.008** ^∗^
PLR	0.597	0.530–0.663	167.5	57.3%	63.9%	0.212	**.008** ^∗^
ESR	0.834	0.791–0.877	34.5	67.2%	91.7%	0.589	**<.001** ^∗^
CRP	0.889	0.858–0.917	7.5	76.2%	91.7%	0.679	**<.001** ^∗^
ESR+NLR	0.836	0.794–0.878	35.5	71.6%	88.9%	0.605	**<.001** ^∗^
ESR+PLR	0.681	0.617–0.744	200.5	66.7%	66.7%	0.334	**<.001** ^∗^
ESR+NLR+PLR	0.681	0.617–0.744	202.5	66.7%	66.7%	0.334	**<.001** ^∗^
CRP+NLR	0.874	0.843–0.905	10.5	77.7%	88.9%	0.666	**<.001** ^∗^
CRP+PLR	0.665	0.602–0.729	191.5	57.9%	72.2%	0.301	**<.001** ^∗^
CRP+NLR+PLR	0.664	0.601–0.727	194.5	58.1%	72.2%	0.303	**<.001** ^∗^

AUC = area under curve, CI = confidence interval, CRP = C-reactive protein, ESR = erythrocyte sedimentation rate, NLR = neutrophil/lymphocyte ratio, PLR = platelet/lymphocyte ratio, RA = rheumatoid arthritis.

∗ Statistically significant difference.

## 4 Discussion

The inflammatory response can promote the formation of pannus in the joint, which is the major cause of joint damage.^[23]^ Although a deeper understanding of RA has been developed due to more in-depth studies in recent years, it remains a challenge to assess the severity of inflammatory activity in patients with RA.^[24]^ The current RA evaluation still has some limitations. For example, ESR, CRP, RF, DAS and the clinical disease activity index are often at cutoff thresholds and are overlooked in patients with low disease activity, but those patients are still at risk of synovial inflammation and progressive joint damage. In a previous study, the data also demonstrated that bone and joint damage can continue to progress in certain patients due to persistent synovial inflammation, even in clinical remission.^[25]^ Thus, it is critical to accurately evaluate the severity of inflammation.

In recent years, the NLR, PLR and LMR have served as novel nonspecific inflammatory markers that are attractive to investigators in different fields, including inflammatory diseases (i.e., ankylosing spondylitis, active familial Mediterranean fever, Henoch–Schonlein purpura and RA).^[15,26–28]^ The NLR represents two compartments of the immune system. Neutrophils representing the innate system are at the front line of the defense system and are responsible for the production of lytic enzymes, oxygen free radicals, and cytokines. Lymphocytes represent the adaptive system. The persistent accumulation of lymphocytes at the sites of inflammatory joints might result in a decreased lymphocyte count in peripheral blood in patients with RA so that peripheral lymphopenia and the gradual increase in neutrophil count have often been noted with the progression of RA. Monocytes and platelets also have regulatory effects on the immune system, have an active role in inflammation and are involved in the production of cytokines. Thus, the changes caused by inflammation in neutrophils, lymphocytes, monocytes and platelets have turned the NLR, PLR, and LMR into inflammatory markers.

Our present study assessed the relationships among the NLR, PLR, and LMR and disease activity in patients with RA. According to the DAS28, the NLR and PLR were lower in patients in remission, with a score of less than 2.6, than in patients with active disease, which is consistent with previous studies.^[16–20]^ Du's study^[17]^ suggested that the LMR is an important inflammatory marker that could be used to identify disease activity in patients with RA, but no significant differences were identified in the LMR between patients in remission and patients with active disease in our present study.

A weak positive correlation was observed between the CRP, ESR and DAS28 score used to assess disease activity and the NLR and PLR. Receiver operating characteristic analysis showed that CRP was the best laboratory diagnostic indicator for disease activity in RA, with an AUC of 0.889, a diagnostic sensitivity of 76.2% and a specificity of 91.7%. For the NLR (the sensitivity and specificity were 31.8% and 77.8%, respectively) and PLR (the sensitivity and specificity were 57.3% and 63.9%, respectively), the diagnostic values were all lower than the corresponding values for CRP and ESR. These data indicated that the NLR and PLR were less valuable than CRP and ESR for differentiating inactive RA patients from active patients and combining NLR or PLR also cannot significantly improved the diagnostic value of ESR and CRP, which means that they may not be useful indicators in detecting disease activity due to low sensitivity and specificity.

Inevitably, our study still has some potential limitations. First, our present study was a retrospective and single-center study. There is a huge difference between those with high disease activity and the others that may cause selection bias. Therefore, a multicenter prospective study is required in the future. Second, due to the relatively small number of patients included in the study, a larger number of patients could enable better statistical evaluation. Third, we did not explore the relationship of hematological markers with indices other than DAS28, such as the CDAI or SDAI.

Taken together, although the patients with disease activity presented higher NLR and PLR compared to patients in remission, both the NLR and PLR showed lower discrimination power than CRP and ESR in detecting disease activity and combining NLR or PLR cannot significantly improved the diagnostic value of ESR and CRP. Thus, NLR, PLR and LMR may not be useful independent diagnostic or complementary markers for disease activity in patients with RA.

## Author contributions

**Conceptualization:** Wang Lijuan, Liang Chaoyang, Yang Ju.

**Formal analysis:** Wang Lijuan, Zhou Yuting.

**Investigation:** Wang Lijuan.

**Methodology:** Wang Lijuan, Zhou Yuting, Liang Chaoyang, Yang Ju.

**Validation:** Liang Chaoyang, Yang Ju.

**Writing – original draft:** Wang Lijuan, Zhou Yuting.
